# Segmental Tissue Resistance of Healthy Young Adults during Four Hours of 6-Degree Head-Down-Tilt Positioning

**DOI:** 10.3390/s23052793

**Published:** 2023-03-03

**Authors:** Todd J. Freeborn, Shelby Critcher, Gwendolyn Hooper

**Affiliations:** 1 Department of Electrical and Computer Engineering, College of Engineering, The University of Alabama, Tuscaloosa, AL 35487, USA; 2 Capstone College of Nursing, The University of Alabama, Tuscaloosa, AL 35487, USA

**Keywords:** bioimpedance, tissue resistance, segmental resistance, fluid-shifts, emulated microgravity, head-down-tilt

## Abstract

(1) Background: One effect of microgravity on the human body is fluid redistribution due to the removal of the hydrostatic gravitational gradient. These fluid shifts are expected to be the source of severe medical risks and it is critical to advance methods to monitor them in real-time. One technique to monitor fluid shifts captures the electrical impedance of segmental tissues, but limited research is available to evaluate if fluid shifts in response to microgravity are symmetrical due to the bilateral symmetry of the body. This study aims to evaluate this fluid shift symmetry. (2) Methods: Segmental tissue resistance at 10 kHz and 100 kHz was collected at 30 min intervals from the left/right arm, leg, and trunk of 12 healthy adults over 4 h of 6° head-down-tilt body positioning. (3) Results: Statistically significant increases were observed in the segmental leg resistances, first observed at 120 min and 90 min for 10 kHz and 100 kHz measurements, respectively. Median increases were approximately 11% to 12% for the 10 kHz resistance and 9% for the 100 kHz resistance. No statistically significant changes in the segmental arm or trunk resistance. Comparing the left and right segmental leg resistance, there were no statistically significant differences in the resistance changes based on the side of the body. (4) Conclusions: The fluid shifts induced by the 6° body position resulted in similar changes in both left and right body segments (that had statistically significant changes in this work). These findings support that future wearable systems to monitor microgravity-induced fluid shifts may only require monitoring of one side of body segments (reducing the hardware needed for the system).

## 1. Introduction

During space travel, astronauts are subjected to extreme environmental conditions in comparison to conditions on the Earth’s surface. These extreme conditions include high radiation exposure and microgravity. Both of these conditions cause a range of adaptations in the human body [1]. These adaptations affect the musculoskeletal system (muscle atrophy and loss of bone mineral density), renal system (kidney stone formation), and the vision system (vision impairment). One effect of microgravity on the human body is the redistribution of fluid due to the removal of the hydrostatic gravitational gradient [1,2]. Without this gradient, fluid redistributes from the legs toward the chest and head [1,3]. This is referred to as a cephalad fluid shift.

From a query of 300 astronauts, Mader et al. reported that approximately 29% of astronauts on short-duration missions and 60% of astronauts on long-duration missions experienced a degradation in vision (both distant and near) [4]. Their hypothesis is that optic nerve and ocular changes may be the result of the cephalad fluid shifts induced by microgravity exposure. Nelson et al. reported in their review of microgravity-induced fluid shifts and ophthalmic changes that permanent loss of visual acuity is one of the highest perceived medical risks of a long spaceflight [5]. With microgravity-induced fluid shifts expected to be the source of severe medical risks combined with the expectation of space missions increasing in duration, it is critical to have more knowledge of how fluid shifts impact various body systems, how to monitor fluid shifts, and how to develop counter-measures to reduce their impact on astronauts during and after exposure to microgravity.

One measurement methodology that has been investigated for monitoring and quantifying fluid shifts is the electrical impedance of biological tissues [6,7,8,9,10]. The passive electrical impedance of tissues (often referred to as tissue bioimpedance) is related to the tissue fluid, type, structure, and geometry. These measurements are collected by exciting the tissue with a low amplitude electrical current (*I*, below the perception threshold of an individual) and measuring the voltage response (*V*) of the tissue from which the impedance is calculated (Z=V/I). These measurements are non-invasive and non-ionizing, which make them an attractive monitoring solution. Changes in physiology features are expected to alter the tissue impedance and potentially serve as a biomarker of that underlying mechanism (e.g., fluid shift, tissue damage, tissue swelling). For fluid shift-related applications, the change in the amount (decrease or increase) of fluid within a region of the body is expected to alter the resistance of that body segment (increase or decrease, respectively). Therefore, monitoring the electrical impedance of multiple body segments, such as the arms, legs, and trunks has the potential to reflect fluid distribution (and redistribution).

The use of bioimpedance to measure fluid redistribution (also referred to as impedance plethysmography) for space applications have been reported as early as 1987 with Montgomery providing an overview of 4 specific studies investigating segmental fluid shifts induced by horizontal or −6° head-down bed rest [6]. Montgomery concluded that calf volume changes due to horizontal or −6° head-down bed rest were immediate, progressive, and uniform across the study participants, not indicative of other segmental changes with time or across subjects. Further, the impedance-derived volumes were highly corrected with anthropometric data [6]. Additional studies utilized bioimpedance monitoring to compare fluid shifts during horizontal bed rest between men and women (reporting that cephalad shifts between men and women may be different) [11] and to assess hydration and body composition of cosmonauts onboard the International Space Station [10]. Additionally, studies of segmental fluid redistribution from transitions in body positions (e.g., sit to stand, sit to supine, etc.) have utilized tissue impedance to measure these alterations [8,12,13,14]. From this body of knowledge, an emerging trend is that segmental leg resistance changes with body positions as a result of the change in the hydrostatic gravitation gradient. More specifically, the observed trend is that laying in a supine or head-down tilt position increases the segmental leg resistance and is attributed to the movement of fluid out of this body segment. Support for this trend is provided by (1) Fenech and Jaffrin, who reported average increases of 18.16% in extra-cellular leg resistance of 15 study participants after 30 min in a supine position [8], (2) Zhu et al., who reported average increases of 13.1% in extra-cellular leg resistance of 6 study participants after 30 min in a supine position [13], and (3) Berg, who reported an approximately 11% increase in calf resistance of 7 participants after 120 min in a supine position [12]. Now, while there are variations across these studies, in terms of electrode configuration and measurement frequency the trend of increasing leg resistance in the supine body position is consistent. The assumption that these increases result from the cephalad fluid shifts supports the application of bioimpedance sensing for monitoring segmental fluid shifts and modeling total body fluid distribution for persons in a microgravity environment.

One limiting factor toward the adoption of bioimpedance sensing in microgravity environments is that measurements have traditionally required laboratory [11] instruments or portable equipment [15]. These instruments prevent unobtrusive and continuous monitoring. Recently, however, the increasing availability of low-cost commercial sensors with bioimpedance capabilities [16] is facilitating the development of wearable systems [17,18] to collect tissue bioimpedance continuously. Wearable, non-invasive, and unobtrusive bioimpedance sensors could accurately quantify the real-time, in-space dynamics of fluid shifts. These sensors could collect data from persons in microgravity conditions to improve fluid models of the human body and even be used to evaluate countermeasures to cephalad fluid shifts. However, further information is needed to determine the necessary requirements for wearable systems focused on this application. Further information to support the development of wearables for this application includes identifying the necessary body segments to monitor, the necessary resolution and measurement range, and the optimum electrode locations. While the arm, leg, and trunk segments have been monitored in previous studies [8,11,13,14], only a single side of the body has been measured. An implied assumption is that fluid shifts are symmetrical due to the bilateral symmetry of the body. That is, the right and left segments have the same fluid shifts in response to changes in body position or gravitational gradients. However, this assumption has not been validated, which provided the motivation for this study. Specifically, to evaluate the question: are there differences between the resistance alterations of the right and left body segments in response to short-term (4 h) of 6° head-down tilt (HDT) body positioning? The following sections outline methods for collecting segmental tissue resistance from study participants, the methods for analysis, analysis results, and the discussion of these results in the context of previous fluid shift studies and the implications for wearable systems.

## 2. Methods

To evaluate the study question, segmental tissue resistance from 7 to 100 kHz was collected from 6 body segments of healthy, young adults at 30 min intervals while they were in a 6° HDT body position for 4 h. The 6 body segments measured for this study were the left leg, left arm, left trunk, right leg, right arm, and right trunk.

### 2.1. Study Participants

A total of 12 participants were recruited for participation in this study. Inclusion criteria for participation required that participants be between the ages of 18 and 40, have a body mass index (BMI) between 19 and 29, and be recreationally active (defined here as participating in moderate activity for 30 min at least 3 times per week for at least the previous 6 months). Exclusion criteria included any health conditions that may limit their participation in the HDT protocol (e.g., musculoskeletal issues, chronic back pain, head trauma), conditions that may be exacerbated by fluid shifts (e.g., high intracranial pressure, ocular disease, family history of thrombosis), or other chronic health conditions (hepatitis, HIV, diabetes). The average and range (given as the standard deviation) of the age (in years), weight (kg), height (m), and BMI (kg/m^2^) of the participants are provided in Table 1. Prior to their participation in the study, each participant provided their written informed consent. This research and its activities were approved by the University of Alabama’s Institutional Review Board (UA IRB-19-022-ME) prior to the study launch.

### 2.2. Participant Preparation

**Figure 1:**
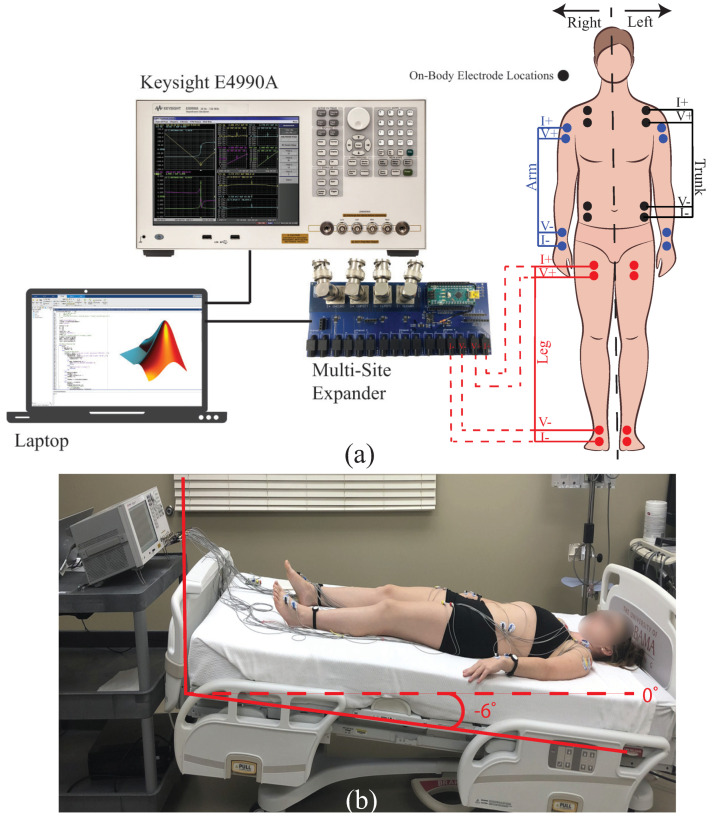
(**a**) Experimental configuration to collect 6 segmental resistance (right/leg arms, trunk, and legs) using Keysight E4990A with multi-site interface expander and (**b**) sample setup of participant during 6° HDT.

Each participant fasted for 12 h prior to their arrival for data collection at the study facilities. After arrival, each participant was asked to void their bladder. Next, the skin surfaces of the electrode sites on the arms, legs, and trunks were cleaned with isopropyl alcohol (70% solution) wipes. After cleaning and allowing the skin site to air dry a total of 24 adhesive Ag/AgCl electrodes (Kendall 133 electrodes) were placed on the left/right trunk, arms, and legs. The specific sites of these placed electrodes are shown in Figure 1a as black, blue, and red dots respectively. After placement of the Ag/AgCl electrodes, participants laid supine on a hospital bed (at 0° incline). Next a Keysight E4990A precision impedance analyzer (Keysight Technologies, Santa Rosa, CA, USA) was interfaced to 6 segments of the participants using 2 m cabling.

### 2.3. Keysight E4990A Impedance Analyzer

The segmental tissue resistance of each body segment at each time point was collected using a Keysight E4990A impedance analyzer. This instrument has been previously investigated for the measurement of biological tissues and reported to be most accurate from 10 to 100 kHz for this application due to the high electrode/tissue interface impedance of this type of measurement [19]. For this reason, measurements were collected from 7 to 100 kHz in this study with this instrument.

The Keysight E4990A utilizes a tetrapolar configuration for impedance measurements. This instrument applies a sinusoidal current at a fixed frequency using two leads (I+, I−) and measures the excitation voltage across a material or device using two additional leads (V+,V−). From the voltage and current, the overall impedance is calculated (Z=V/I) at each of the discrete measured frequencies. For all impedance measurements, the instrument was set not to exceed a current stimulus of 1 mA, to sweep from 7 to 100 kHz, and to use the maximum measurement time. To configure the maximum measurement time the internal instrument setting MEAS was set to 5. This setting can have values from 1 (fast) to 5 (precise). Using MEAS = 5 each impedance measurement in the range from 1 to 100 kHz requires 0.2 s per frequency.

To measure 6 body segments a custom Keysight E4990A interface was utilized. This interface increased the number of tetrapolar electrode configurations that can be measured (without manually changing wires/leads) from 1 to 8. The interface was a custom printed circuit board (PCB) realization of analog multiplexors to electronically control the routing of the Keysight E4990A tetrapolar signals (I+, I−, V+, V−) to 1 of 8 sets of mechanical headers. For this study, 6 of the available 8 sets of headers were wired to participant body segments. This configuration of the instrument, custom PCB, and wiring to a body segment is shown in Figure 1a. Both the PCB interface and Keysight E4990A were controlled during data collection by a connected laptop computer using MATLAB 2022a (v9.12), which automated the process. For data collection, commands were transmitted using the serial port from MATLAB to the custom PCB (which had an on-board Arduino Uno for decoding serial communications) to (1) configure on-board analog multiplexors for appropriate routing of the test signals; (2) initiate measurement sweeps by the Keysight E4990A (using the USB interface to transmit SCPI commands for instrument control); (3) transfer the collected impedance data to MATLAB (using USB after receiving the appropriate SCPI commands) and; (4) storing the data as native MATLAB workspace variables (.mat) for post-processing. A custom MATLAB library was developed that implemented the SCPI commands for the Keysight E4990A instrument. The command syntax for this instrument is outlined in the Keysight E4990A programming guide. To compensate for the effects of the interface PCB and wiring the open/short/load compensation procedure was applied with the Keysight E4990A prior to measurements of the study participants

Upon completion of the instrument setup, a baseline measurement was collected from each participant while in a 0° supine position (referred to in this work as the pre-tilt measurement). Next, the hospital bed was adjusted so the participant experienced a 6° head-down tilt. A photo of this setup for a single participant in the 6° HDT position is provided in Figure 1b. After tilting, impedance measurements were collected from each segment at 30 min intervals for 4 h. This yielded measurements at 9 time points (0, 30, 60, 90, 120, 150, 180, 210, and 240 min). Prior to each measurement participants were asked to remain motionless. Collection of the resistance data from all 6 body segments required approximately 2 min at each protocol time point. Between measurements, participants were asked to limit their movements but were allowed to reposition themselves (without standing or sitting up) as necessary for comfort. After 4 h, the hospital bed was returned to 0° and the participant slowly transitioned from supine, to sitting, to standing as they were comfortable.

### 2.4. Data Post-Processing

**Figure 2:**
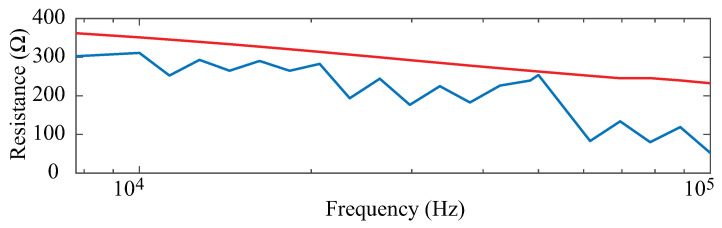
Sample of participant segmental leg resistance with (blue) and without (red) artifacts from visual data inspection prior to analysis.

The collected resistance data were processed in MATLAB to assess quality and limit datasets with artifacts (e.g., data that was not representative of the tissue) from potential equipment errors or measurement conditions from affecting processing and interpretation. The resistance data from all participants at each time point were visually inspected to identify potential artifacts. An example resistance dataset that was collected from the left leg of a participant with suspected artifacts is shown in Figure 2 as a blue line. The left leg resistance in Figure 2 does not show a consistent trend of decreasing resistance with increasing frequency that is expected for tissue measurements. For comparison, a dataset that does follow the expected is shown in Figure 2 as a red line. This dataset is the right leg resistance of the same participant measured at the same time point. The visually jagged trend of the left leg data could be a result of muscle contractions/movement during measurement of this body segment. Kitchin and Freeborn previously reported increases in resistance up to 6.75 Ω increase in a stepped-sine bioimpedance measurement (also utilized in this work) from contraction during their measurement of the biceps tissues [20]. The observed alterations of the left leg resistance in Figure 2 are similar to those reported for a contraction event and were not observed in the consecutive measurements before or after this time point. This supports that this specific measurement was not representative of the tissue and was labeled an artifact. Participants with artifacts in one or more of their collected datasets were removed from further analysis. Based on this, the exclusion criteria data from 8 of the 12 participants were included for analysis.

## 3. Results

**Figure 3:**
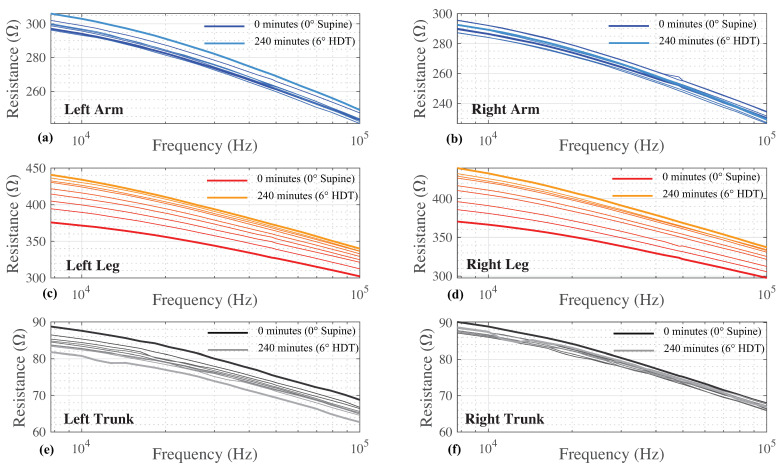
Segmental tissue resistance (from 7 to 100 kHz) of (**a**) left arm, (**b**) right arm, (**c**) left leg, (**d**) right leg, (**e**) left trunk, and (**f**) right trunk of a single study participant collected at 30 min intervals while the participant was in the HDT position. The darkest and lightest lines indicate the first and last measurements, respectively, through this protocol.

The multi-frequency (7 to 100 kHz) resistance for all time points and body segments from a single participant is provided in Figure 3 as a sample of participant data included for analysis. The 8 sets of resistance data for each body segment (left and right arm/leg/trunk) are shown in Figure 3a–f. Different color shades represent measurements collected at different time points with the darkest shade representing data at 0 min and the lightest shade at 240 min. Each segmental resistance shows a decreasing resistance value with increasing frequency, which is typical of biological tissues. For this particular participant, there is a general trend of increasing segmental resistance (at all measured frequencies) between the measurements at 0 min and 240 min for the arms and legs, with the opposite trend (decreasing resistance) for the trunk.

For further analysis resistances at two discrete frequencies, 10 kHz and 100 kHz, were utilized. “Low” and “high” frequency resistances are often attributed to extra-cellular and total tissue fluids, respectively, supporting their independent analysis here. The median 10 kHz and 100 kHz resistances of all participants segmented (left/right leg, arm, and trunk) are provided in Table 2 and Table 3, respectively.

### 3.1. Statistical Analysis

To evaluate if there were statistically significant differences in the 10 kHz and 100 kHz resistances of each segment resulting from the length of time in the HDT position, a Friedman test (SPSS Statistics) was applied to the repeated measures data. For this testing, the dependent variable was segmental resistance and the independent variable was time. The testing of the 10 kHz and 100 kHz right and left leg segments rejected the null hypothesis supporting that there were statistically significant differences of median resistance at the different time points during the HDT protocol (10 kHz left leg: $\chi^{2}\left(8\right)=56.867$, p<0.0005; 10 kHz right leg: $\chi^{2}\left(8\right)=62.067$, p<0.0005, 100 kHz left leg: $\chi^{2}\left(8\right)=38.1$, p<0.0005, 100 kHz right leg: $\chi^{2}\left(8\right)=62.033$, p<0.0005). The 10 kHz and 100 kHz right trunks were also statistically different at the different time points (10 kHz right trunk: $\chi^{2}\left(8\right)=16.0$, p=0.042; 100 kHz right trunk: $\chi^{2}\left(8\right)=25.4$, p=0.001).

Pairwise comparisons were performed (SPSS Statistics) with a Bonferroni correction for multiple (8) comparisons between the measurements at 0 min and all other time points. The 10 kHz leg resistance (for both left and right segments) was statistically significantly different (p<0.05) at 120, 150, 180, 210, and 240 min compared to the baseline value. These specific time points are denoted by the ${}^{*}$ symbol in Table 2. The median 10 kHz values between 0 min and 240 min of the left/right legs had an increase of 34.5 Ω and 38.3 Ω, respectively. The 100 kHz leg resistances (for both left and right segments) were also statistically significantly different (p<0.05) at 90, 120, 150, 180, 210, and 240 min compared to the baseline value. These specific time points are denoted by the ${}^{*}$ symbol in Table 3. The median 100 kHz values between 0 min and 240 min of the left/right legs had an increase of 21.1 Ω and 21.8 Ω, respectively. From the pairwise comparisons for both the segmental arms and trunk resistances there were no statistically significant differences between baseline and later time points.

### 3.2. Relative Differences

**Figure 4:**
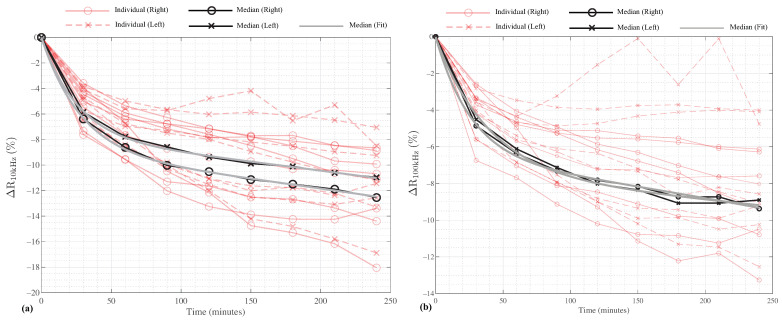
Relative difference of (**a**) 10 kHz and (**b**) 100 kHz segmental resistances from left (x) and right (o) legs of study participants over 240 min of HDT with median (black) and fit (gray) values.

The relative differences between the 10 kHz and 100 kHz segmental resistances from t=30 min to t=240 min (in comparison to the baseline at t=0) were calculated using:

(1) $$\Delta R=\frac{R_{x}-R_{0}}{R_{0}}\times 100$$

where ΔR is the relative difference, $R_{0}$ is the resistance at t=0 min, and $R_{x}$ is the resistance at time point *x*, where x=30, 60, 90, ⋯, 240 min. The calculated relative differences for the participants are given in Figure 4a,b for the 10 kHz and 100 kHz resistances, respectively, as red lines. For reference, the median values (previously reported in Table 2 and Table 3) are also shown as solid black lines for both the left and right segments.

The median resistances show a decreasing rate of change with increasing time in the HDT position. This implies that there are potentially two-decay modes (e.g., different mechanisms governing the rates of change). To model this behavior, the median datasets were fit to the two-term exponential function given by:

(2) $$\Delta R\left(t\right)=a_{1}e^{t/\tau_{1}}+a_{2}e^{t/\tau_{2}}$$

where $a_{1,2}$ are the exponential coefficients, $\tau_{1,2}$ are the exponential time constants, and *t* is the time (in min). Using the curve-fitting interactive application in MATLAB, the coefficients that best fit (2) to the median segmental leg resistance are:

(3) $$\begin{array}{ccc} \Delta R_{\mathrm{LL}-10\mathrm{kHz}}\left(t\right) & = & 7.945e^{-t/26.01}-7.956e^{t/735.84}, \end{array}$$

(4) $$\begin{array}{ccc} \Delta R_{\mathrm{RL}-10\mathrm{kHz}}\left(t\right) & = & 9.143e^{-t/28.51}-9.152e^{t/774.59}, \end{array}$$

(5) $$\begin{array}{ccc} \Delta R_{\mathrm{LL}-100\mathrm{kHz}}\left(t\right) & = & 7.542e^{-t/40.02}-7.614e^{t/1267.91}, \end{array}$$

(6) $$\begin{array}{ccc} \Delta R_{\mathrm{RL}-100\mathrm{kHz}}\left(t\right) & = & 6.591e^{-t/26.48}-6.604e^{t/695.41}, \end{array}$$

where the notation LL and RR denote the left leg and right leg, respectively. The functions given by (3) through (6) show very good agreement with the median data, with $R^{2}$ (proportion of the total sum of squares explained by the model) values of 0.999, 0.999, 0.994, and 0.998, respectively. For reference, these functions are also plotted in Figure 4 as solid gray lines. The MATLAB curve-fitting application uses a non-linear least squares approach with the trust-region algorithm. For the fittings in this effort, the default application configuration was utilized. In addition to the good agreement from the $R^{2}$ values, the 95% confidence intervals for each parameter are given in Table 4. Notice that most of the intervals in Table 4 do not include the value zero, confirming that the null hypothesis (e.g., the coefficient is zero) can be rejected for these coefficients. The one exception is the $\tau_{2}$ coefficient for the LL-100 kHz model, which supports that these data can likely be modeled with a single-term exponential function.

### 3.3. Symmetry Differences

A Wilcoxon signed-rank test was conducted to evaluate the study question: Are there differences between the left and right segment resistance alterations induced by 240 min in the 6° HDT position? Only leg resistances were tested because they were the only segments with statistically significant differences during the study protocol. The symmetry differences ($\Delta R_{sym}$) for this test were calculated using:

(7) $$\Delta R_{sym}=\left(R_{240}^{Ri}-R_{0}^{Ri}\right)-\left(R_{240}^{Le}-R_{0}^{Le}\right)$$

where $R_{240}$ and $R_{0}$ denote the resistance measurements at 240 min and 0 min, respectively, and Le or Ri superscripts denote the left or right leg. From the 8 study participants the median $R_{sym}$ values were 2.29 Ω and 2.88 Ω for the 10 kHz and 100 kHz resistances, respectively. From the Wilcoxon signed-rank test, there was not a significant median difference between the 10 kHz (z=−1.26, p=0.208) and 100 kHz (z=−1.68, p=0.093) $R_{sym}$ values. That is, there were no significant differences between the resistance increases of the left and right leg segments.

## 4. Discussion

**Figure 5:**
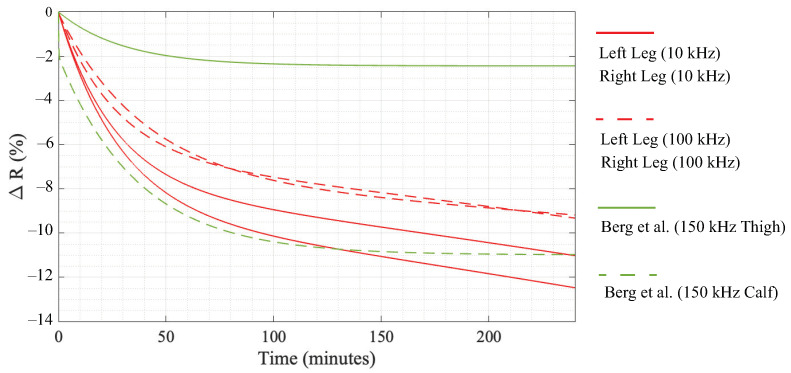
Comparison of the relative 10 and 100 kHz left and right resistance changes from study participants compared to the 150 kHz relative difference between the thigh and calf by Berg et al. [12].

From the results presented in Table 2 and Table 3, the participant’s time in the 6° HDT position resulted in statistically significant increases in the median 10 kHz and 100 kHz resistance. These differences are attributed to the movement of fluid away from the legs and into the trunk of each participant and support that resistance measurements are sensitive to changes in body position (and the resulting fluid redistribution). Comparing the relative differences, the 10 kHz resistance had a larger rate of change until approximately 80 min. This is observed visually in Figure 5 where the 10 and 100 kHz data are given as solid and dashed lines, respectively. This may suggest that the extra-cellular fluid (associated with low-frequency resistance) responds faster to the change in body position. From approximately 80 min to 240 min, both 10 and 100 kHz have similar rates of change. Overall, the relative differences for the 10 kHz segmental leg resistances (in the range from 11% to 12%) were greater than 100 kHz relative differences (approximately 9.5%) after 240 min. It is important to note though that a limitation of this study is the small number of participants (8), which limits the power of this study. Therefore, further research with a greater number of participants is recommended to confirm that the trends reported in this effort are observed.

Overall, the trend of the segmental leg resistance increasing over time aligns with previous reports by Montgomery [6,11] and Berg et al. [12] who similarly measured segmental leg resistances in adults during bed rest. Berg et al. reported relative differences of thigh and calf 150 kHz resistance given by $\Delta R_{\mathrm{thigh}}\left(\%\right)=-2.44(1-e^{-t/30.3}$ and $\Delta R_{\mathrm{calf}}\left(\%\right)=-2.02\left(1-e^{-}t/0.138\right)-8.97\left(1-e^{-t/36.8}\right)$. The relative differences modeled by these equations are plotted in Figure 5 as solid and dashed green lines, respectively [12]. The median relative differences of our study lie between Bergs reports of calf and thigh resistance (but are similar in magnitude and rate of change for t<120 min). Specifically, the calf resistance reported by Berg et al. had a relative increase of approximately 11% after 120 in a supine position, which is similar to the approximately 8% increase and 9–11% increase of the 100 and 10 kHz resistance at 120 min shown in Figure 5 for the participants in this study. The differences in magnitude between studies are attributed to differences in the measurement setups. Berg et al. measured the leg as two segments (with greater changes in the calf than thigh) in comparison to one segment in this work. Therefore, our results capture changes in both calf/thigh servings as the “average” in comparison to those of Berg et al. Berg et al. only measured participants for 120 min and also only had participants in a supine position. This lower period of measurement and different body positions are expected to be the source of their models deviating from the trends reported here. Yadollahi et al. also reported statistically significant decreases in segmental leg volume (estimated from segmental leg resistance) after 90 min in the supine body position [14]. Yadollahi et al. reported that the changes were well modeled by exponential functions (but complete details of those functions and the raw resistance were not provided limiting direct comparison here). Overall, these combined studies support that the relative changes in segmental resistance are captured by exponential models with similar rates of change across studies and conditions.

Of particular interest regarding the segmental changes in this work is that the segmental trunk resistance did not show statistically significant differences at any time point in HDT compared to the pre-tilt measures. An unexpected finding as the fluid redistribution away from the legs while in HDT is expected to flow to the trunk and decrease the segmental resistance. In comparison to previous studies, Montgomery reported increases in segmental volume change after short-term (4 h) bed rest estimated from segmental trunk resistances but these increases were not statistically significant. Fenech and Jaffrin also reported no statistically significant changes in extra-cellular resistance (an alternative proxy for low-frequency resistance) for the trunk after 30 min supine [8]. However, Yadollahi et al. did report statistically significant differences in segmental trunk volumes of men and women in supine positions after 90 min [14]. In comparison to this work and the works of Montgomery [11], and Fenech and Jaffrin [8], the trunk was measured by Yadollahi et al. as two segments (thorax, abdomen). In this configuration thorax values had a statistically significant increase in fluid (e.g., a decrease in resistance) in both men and women but no increase in abdomen values [14]. This may suggest that the trunk should be monitored as multiple segments to capture fluid redistribution and why changes were not observed here.

Reviewing the last measured segment (arm), there were no statistically significant changes in the participants of this study. Similar to the trunk this was unexpected as the HDT position was expected to induce a shift of fluid from the arms towards the trunk/neck (resulting in a resistance increase of the arm segment). Fenech and Jaffrin reported a statistically significant increase in extra-cellular resistance (e.g., a proxy for low-frequency resistance) in participants during their 30 min supine protocol, supporting that supine positions did cause a change in arm fluid/resistance. The lack of changes observed in this study may be caused by participants being allowed to move and reposition their arms throughout the HDT protocol (and only required to be still during measurements). The muscle contractions during movement may reduce or counter the expected cephalad fluid shifts.

Focusing on the main study question the comparison of the right and left segmental leg resistances, there were no statistically significant differences in either 10 or 100 kHz measurements based on the side of the body measured after 240 min of 6° HDT. These results support the implied assumption of earlier studies that fluid shifts would be similar for both left and right bodies (suggesting that only a single side of the body can be measured to estimate segmental fluids).

For the future design of wearable bioimpedance systems aimed at monitoring segmental fluid shifts in microgravity environments, the results of this study and previous works [6,8,11,12] suggest that systems can measure a single side of the body (left or right) and that the legs and trunk should be the segments of focus. This can potentially reduce the amount of hardware and processing required of a wearable system. Further, accurately estimating trunk fluid distributions will likely require monitoring the trunk as multiple segments (e.g., thorax, abdomen). Future efforts will also be necessary to explore the number of trunk "segments" for this application and investigate measuring the neck resistance (which has not been measured in the previous studies) as an additional segment to quantify fluid shifts towards the head. The exponentially decreasing relative difference observed at both 10 kHz and 100 kHz resistance for the segmental leg measurements suggests it may be possible to further simplify the requirements of a wearable bioimpedance system to monitor only a single frequency, potentially only 10 kHz (which corresponded to the larger rate of change observed in this study). Though further research is needed to confirm if a single frequency is appropriate for capturing changes across longer-time scales. It may be that intra-cellular fluid (associated with higher frequencies) may become more significant at longer time scales and require monitoring of both low and high-frequency segmental resistance.

Another important aspect of the adoption of wearable bioimpedance systems for fluid shift monitoring is the effect of movement and muscle contraction on tissue resistance. Muscle contractions were reported by Kitchin and Freeborn to increase bicep tissue resistance up to 6.75 Ω when curling a 15 lb dumbbell [20]. Therefore, muscle contractions due to activity could lead to incorrect interpretation of fluid shifts in resistance data. Consider that the median left/right 10 kHz segmental arm resistance of this study, given in Table 2, had a difference of 3.8 Ω and 3.6 Ω, respectively, between the measurements at 0 and 240 min. While there was no statistically significant difference between these values in this study, additional increases of 6 Ω or greater due to leg muscle contractions have the potential to alter the study results and be interpreted as a fluid shift. Therefore, systems aimed at providing continuous, real-time monitoring will require processing techniques to identify artifacts (such as contraction events) and remove or correct them towards accurately estimating fluid distributions across conditions. Another important consideration is that studies of fluid shifts to date (including this study) have required participants to remain motionless. However, this is not realistic of the expected use conditions by astronauts in microgravity environments. In fact, fluid shift monitoring during activities and evaluation of counter-measures (such as lower-body negative pressure [21]) will require monitoring during movement. Future studies should explore sensing modalities that complement bioimpedance to capture movement (e.g., inertial measurement units) and muscle activity (e.g., surface electromyography). The additional sensing modalities are expected to increase the accuracy of fluid estimates by helping identify when motion-related artifacts are introduced to the resistance data, which can be used to either remove or correct degraded data. These future efforts are expected to help advance solutions to provide comprehensive fluid shift monitoring to support the personalized and continuous monitoring of astronauts in microgravity conditions.

## 5. Conclusions

From participation in 6° HDT positioning, the segmental leg tissue resistance of the study participants had statistically significant increases first observed at 120 and 90 min for the 10 and 100 kHz measurements, respectively (attributed to the cephalad fluid shift induced by the HDT protocol). These median increases were approximately 11% to 12% for the 10 kHz resistance and 9% for the 100 kHz resistance; they align with previous studies on fluid shifts due to bed rest. In this effort, no statistically significant changes occurred in the segmental arm or trunk resistance. Comparing the left and right segmental leg measurements, there were no statistically significant differences in the resistance changes based on the body side. This evidence shows that future wearable systems to monitor microgravity-induced fluid shifts may only require monitoring one side of body segments (reducing the hardware needed for the system).

## Figures and Tables

**Table 1:** Demographics of study participants (N=12).

Age (years), range 18–31	25.1 ± 3.40
Weight (kg), range 54.60–90.52	72.76 ± 10.53
Height (m), range 1.64–1.94	1.78 ± 0.09
BMI (kg/m^2^), range 19–27.3	23.17 ± 2.63
Sex	
Male, no. (%)	8 (67)
Female, no. (%)	4 (33)

**Table 2:** Median 10 kHz resistance of left and right legs, arms, and trunk segments of the study participants.

Segment	Side	10 kHz Resistance (Ω) at the Time Point (in min):								
		0	30	60	90	120	150	180	210	240
Leg	Left	314.7	333.1	339.2	341.7	344.2 *	345.8 *	346.5 *	348.1 *	349.2 *
	Right	305.3	324.8	331.6	335.9	337.4 *	339.3 *	340.4 *	341.6 *	343.6 *
Arm	Left	276.2	280.5	283.7	283	287.1	282.8	286	282.8	280
	Right	279.9	282.8	284.8	286.8	288	285.6	284.8	285.4	283.5
Trunk	Left	82.6	81.3	80.5	80.4	81.3	80.2	80.6	80.4	79.4
	Right	79.8	79.8	80.1	79.3	80.1	80.8	80.5	80.7	80.6

* denotes statistically significantly different (*p* < 0.05) compared to *t* = 0 (pre-tilt) measurement.

**Table 3:** Median 100 kHz resistance of left and right legs, arms, and trunk segments of the study participants.

Segment	Side	100 kHz Resistance (Ω) at the Time Point (in min):								
		0	30	60	90	120	150	180	210	240
Leg	Left	237	247.7	251.5	253.9 *	256 *	256.8 *	258.5 *	258.5 *	258.1 *
	Right	232.6	243.9	247.4	249.6 *	250.8 *	251.6 *	252.9 *	252.9 *	254.4 *
Arm	Left	222.8	224.6	225.7	225.5	223.4	219.7	218.7	216.6	214.9
	Right	219.8	222.5	223.2	223.1	222.7	221.4	221.2	221	220
Trunk	Left	62.4	61.4	60.6	60.5	61.5	60.6	60.9	60.7	59.7
	Right	58.7	58.7	58.4	57.9	59	59.5	59.3	59.2	59.3

* denotes statistically significantly different (*p* < 0.05) compared to *t* = 0 (pre-tilt) measurement.

**Table 4:** 95% Confidence intervals of coefficients from the fitting of median segmental leg resistances to the two-term exponential function.

Fit	$a_{1}$	$a_{2}$	$\tau_{1}$	$\tau_{2}$
LL-10 kHz	[7.424,8.466]	[−8.398,−7.514]	[−30.81,−22.50]	[604.59,939.85]
RL-10 kHz	[8.588,9.698]	[−9.641,−8.663]	[−33.07,−25.06]	[636.94,988.14]
LL-100 kHz	[5.556,9.527]	[−9.576,−5.651]	[−77.70,−26.95]	[−2207.5,492.6]
RL-100 kHz	[5.949,7.233]	[−7.151,−6.058]	[−34.42,−21.52]	[533.05,999.00]

## Data Availability

The data presented in this study are available upon request from the corresponding author.
